# Targeting colony stimulating factor-1 receptor signalling to treat ectopic pregnancy

**DOI:** 10.1038/s41598-020-72785-y

**Published:** 2020-09-24

**Authors:** S. Furquan Ahmad, W. Colin Duncan, Lisa L. Campbell, Robyn E. Beaty, Magda Koscielniak, Frances Collins, Philippa T. K. Saunders, Andrew W. Horne

**Affiliations:** 1 MRC Centre for Reproductive Health, University of Edinburgh, Queen’s Medical Research Institute, Edinburgh, EH16 4TJ UK; 2 Edinburgh Medical School, University of Edinburgh, Edinburgh, EH16 4SA UK; 3 Centre for Inflammation Research, University of Edinburgh, Queen’s Medical Research Institute, Edinburgh, EH16 4TJ UK

**Keywords:** Cell migration, Endocrine system and metabolic diseases, Reproductive disorders, Endocrine reproductive disorders

## Abstract

1–2% of pregnancies are ectopic, the majority implanting in the Fallopian tube. A single, systemic dose of methotrexate, a DNA-synthesis (S phase) inhibitor, has been used since 1991 for outpatient treatment of women with stable EP. However, methotrexate has limited clinical and cost effectiveness, restricting its use to 25–30% of these women. There is an unmet need for better medical treatment for EP. Colony stimulating factor-1 (CSF-1) promotes placentation and creates a pro-inflammatory environment that is fundamental for the maintenance of a normal pregnancy. We hypothesised that CSF-1 is also involved in the placentation and maintenance of an EP. Herein, we demonstrate the immunolocalisation of the CSF-1 receptor (CSF-1R) as well as its ligand (CSF-1) in immortalised first trimester trophoblast cells. We show that a specific CSF-1R kinase inhibitor, GW2580, abolishes CSF-1 induced trophoblast cell proliferation and migration and can be cytotoxic. We then demonstrate the expression of CSF-1R and CSF-1 in the cytotrophoblast and syncytiotrophoblast within ectopic implantation sites from women with EP. Our data suggests that CSF-1 is involved in the survival and proliferation of trophoblast cells in EP. This suggests that pharmacological disruption of CSF-1/CSF-1R signaling axis could be the basis of a new therapeutic for EP.

## Introduction

An ectopic pregnancy (EP) is defined as the implantation of a conceptus outside of the uterus, with > 98% of EPs implanting in the Fallopian tube (FT)^1^. EP represent 1–2% of all pregnancies and are a leading cause of maternal morbidity and mortality in the first trimester of pregnancy^2,3^. Currently, intramuscular methotrexate (MTX), a chemotherapy drug that targets DNA synthesis of trophoblast cells, is the medical management approach most commonly used to resolve EP^4^. However, MTX does not immediately resolve the EP (with an average time to resolution 24–28 days), has a high failure rate (25–30%) and is associated with unpleasant side effects^5^. Moreover, MTX can only resolve selected EP that are small (serum hCG < 3000 IU/L with no fetal cardiac activity) and that have been diagnosed in women that are haemodynamically stable^6^. Consequently, laparoscopic surgical management with removal of the FT with the EP is regarded as the gold standard treatment^7^. However, surgery is not a feasible intervention in low resource settings, has a prolonged recovery time and a risk of surgical complications^8^. Additionally, it can have implications for fertility, notably if the contralateral tube is damaged^9,10^. There is an unmet clinical need for a less toxic, more efficacious and widely accessible EP treatment that leads to more rapid resolution and reduces the requirement for surgery.

Colony stimulating factor-1 (CSF-1) is a glycoprotein which regulates proliferation, differentiation and survival of macrophages^11^, supports growth and proliferation of extravillous trophoblasts^12^, and plays important role in the implantation of embryo in the uterus and subsequent placental growth^13^. This may also be the case in EP as increased macrophage density at the tubal ectopic implantation site has been demonstrated in comparison to the rest of the Fallopian tube^14^. GW2580 is a CSF-1R antagonist and has recently been used in murine models of epithelial ovarian cancer, Alzheimer’s disease, myocardial infarction and spinal cord injury and has been shown to cause reduced infiltration of macrophages and inhibition of cellular proliferation^15–18^. As CSF-1 activity is involved in supporting trophoblast growth we hypothesised that targeting CSF-1 receptor signalling via CSF-1R antagonism could be used as a treatment for EP.

Herein, we demonstrate the expression of CSF-1R as well as CSF-1 in immortalised human first trimester trophoblast cells. We then show that CSF-1R antagonism, using GW2580, can inhibit trophoblast cell proliferation, migration and survival in-vitro. Finally, we confirm the expression of CSF-1R and CSF-1 at human ectopic implantation sites.

## Results

### CSF-1R and CSF-1 are expressed in immortalised first trimester trophoblast cells

**Figure 1:**
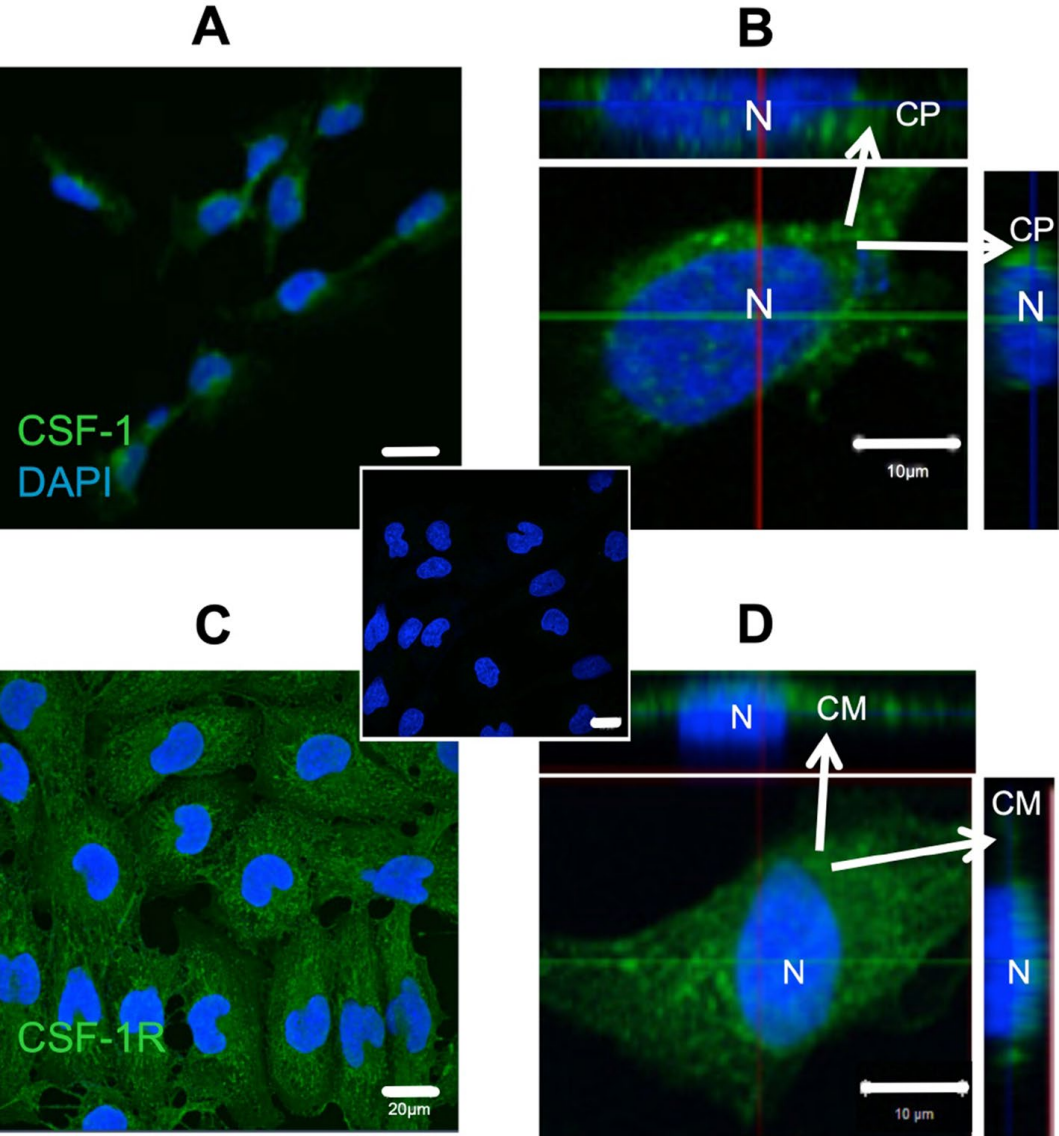
CSF-1R and CSF-1 expression in immortalised first trimester trophoblast cells (SW.71) (**A**) Representative two-dimensional confocal image of SW.71 trophoblast cells immunostained for CSF-1 ligand (green) and nuclei (blue). **(B)** Three-dimensional cross-section image of the SW.71 cell showing localisation of CSF-1 protein in the cytoplasm (CP). **(C)** Representative two-dimension confocal image of trophoblast cells immunostained for CSF-1R (green) and nuclei (blue) in the SW.71 cells. **(D)** Three-dimensional cross-section image of the SW.71 cell showing localisation of CSF-1R in the cell membrane (CM) (Inset) Negative control SW.71 cells with DAPI staining on the nuclei (N) and no immunoreactivity observed. Scale bars A/B/Inset 20 µm and BD 10 µm.

The immortalised first trimester trophoblast cell line, SW.71, expresses CSF-1 protein (Fig. 1A) and it is predominantly localised to the cell cytoplasm (Fig. 1B). As well as the CSF-1 the SW.71 cells also express its receptor CSF-1R (Fig. 1C). The expression of CSF-1R protein was mainly localised to the cell membrane of the SW.71 cells (Fig. 1D). These trophoblasts can both make and respond to CSF-1.

### CSF-1R antagonism reduces proliferation

**Figure 2:**
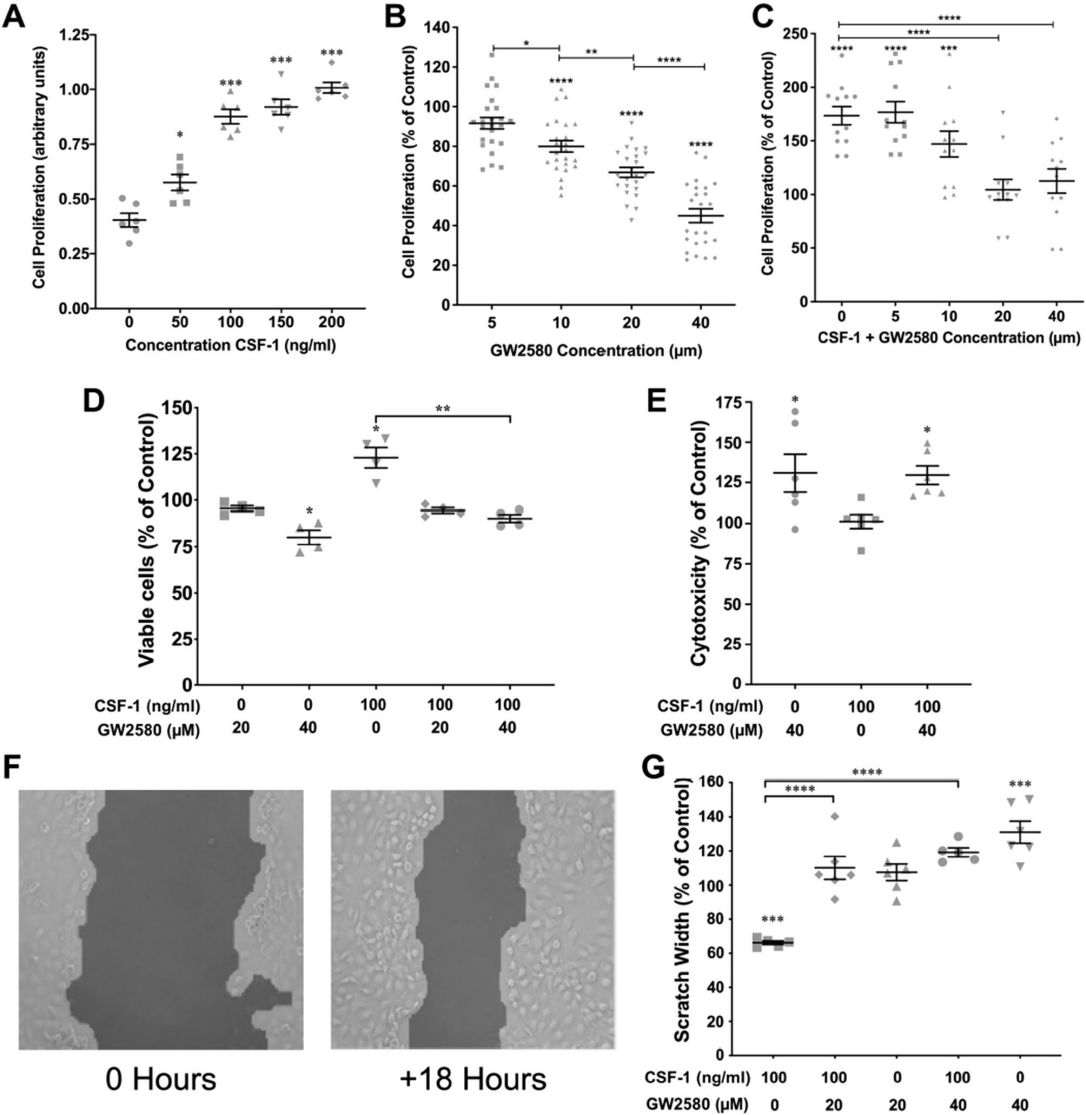
Effect of CSF-1 and CSF-1R antagonism with GW2580 on trophoblast cell proliferation, migration and viability **(A)** Effects of exogenous CSF-1 (50–200 ng/ml) on SW.71 cell proliferation after 48 h exposure. **(B)** Effects of GW2580 (20 and 40 µM) on proliferation as a percentage of vehicle control after 48 h. **(C)** Effects of exogenous CSF-1 (100 ng/ml) with GW2580 (5–40 µM) on as a percentage of vehicle control after 48 h. **(D)** Effect of CSF-1 (100 ng/ml) and GW2580 (20–40 µM) on viable cell number as a percentage of control. **(E)** The effect of 40 µM GW2580 in the presence or absence of CSF-1 (100 ng/ml) of cell cytotoxicity. **(F)** A representative image showing scratch imaged at 0 h and at 18 h using TScratch software. **(G)** Effects of GW2580 (20–40 µM) and exogenous CSF-1 ligand (100 ng/ml) on SW.71 cellular migration after an 18 h incubation. Vehicle (DMSO) treated cells were used as controls and values normalised to the control. *p < 0.05, **p < 0.01, ***p < 0.001, ****p < 0.0001.

As the SW.71 cell line is able to respond to CSF-1 we examined the effects of exogenous CSF-1 on proliferation. CSF-1 caused a dose-dependent increase in cell proliferation (p < 0.0001; Fig. 2A). The maximal effect was seen at a concentration of 100 ng/ml and this concentration was used in subsequent studies. As the SW.71 cell line can also make CSF-1 we examined the effect of the CSF-1 antagonist, GW2580, on proliferation in a dose finding study. After 48 h there was no effect of 5 µM GW2580 but concentrations of 10 µM (p < 0.0001), 20 μM (p < 0.0001) and 40 μM (p < 0.0001) reduced proliferation, when compared to control, in a dose dependent manner (Fig. 2B). The proliferative response to exogenous CSF-1 was blocked by both 20 μM (p < 0.0001) and 40 μM (p < 0.0001) GW2580 (Fig. 2C). We therefore focussed on both 20 µM and 40 µM GW2580 in subsequent experiments. CSF-1 increases proliferation in immortalised first trimester trophoblast cells.

### CSF-1R antagonism can promote cell death

In order to determine whether the cell death was associated with the anti-proliferation effects of CSF-1 antagonism we examined cell health using the ApoTox-Glo triplex assay. Interestingly while 20 µM GW2580 reduced SW.71 proliferation and also could block the proliferative effects of exogenous CSF-1 it was not associated with a reduction in cell viability (Fig. 2D). However, at the 40 µM dose there was a reduction in viability in the absence (p < 0.05) and presence (p < 0.01) of exogenous CSF-1 (Fig. 2D). There was increased cytotoxicity with 40 µM GW2580 (p < 0.05) and this was not overcome by the addition of CSF-1 (p < 0.05; Fig. 2E). CSF-1 promotes cell survival in immortalised first trimester trophoblast cells.

### CSF-1R antagonism decreases cellular migration

We then analysed the effects of GW2580 on cellular migration using a scratch assay. We first optimised the time-point which should be used to study cellular migration, taking measurements of scratch opening (gap between the cell fronts caused by scratch) at 18, 24 and 48 h. We found that differential results could be seen at the 18 h time-point only (Fig. 2F), as after 24 and 48 h of incubation cells tend to fill in the gap of the scratch, even in the presence of inhibitor (data not shown). Exposure to CSF-1 stimulated the migration of the trophoblast cells and scratch width was reduced as compared to vehicle (p < 0.001; Fig. 2G). Exposure to CSF-1 20 µM GW2580 had no effect on cell migration while exposure to 40 µM GW2580 inhibited cell migration, and scratch width was greater, when compared to vehicle control (p < 0.001). However, both 20 µM and 40 µM concentrations inhibited trophoblast migration in the presence of CSF-1, (p < 0.0001; Fig. 2G). CSF-1 increases cell migration in immortalised first trimester trophoblast cells.

### CSF1-R and CSF-1 are expressed in trophoblasts within tubal ectopic implantation sites

**Figure 3:**
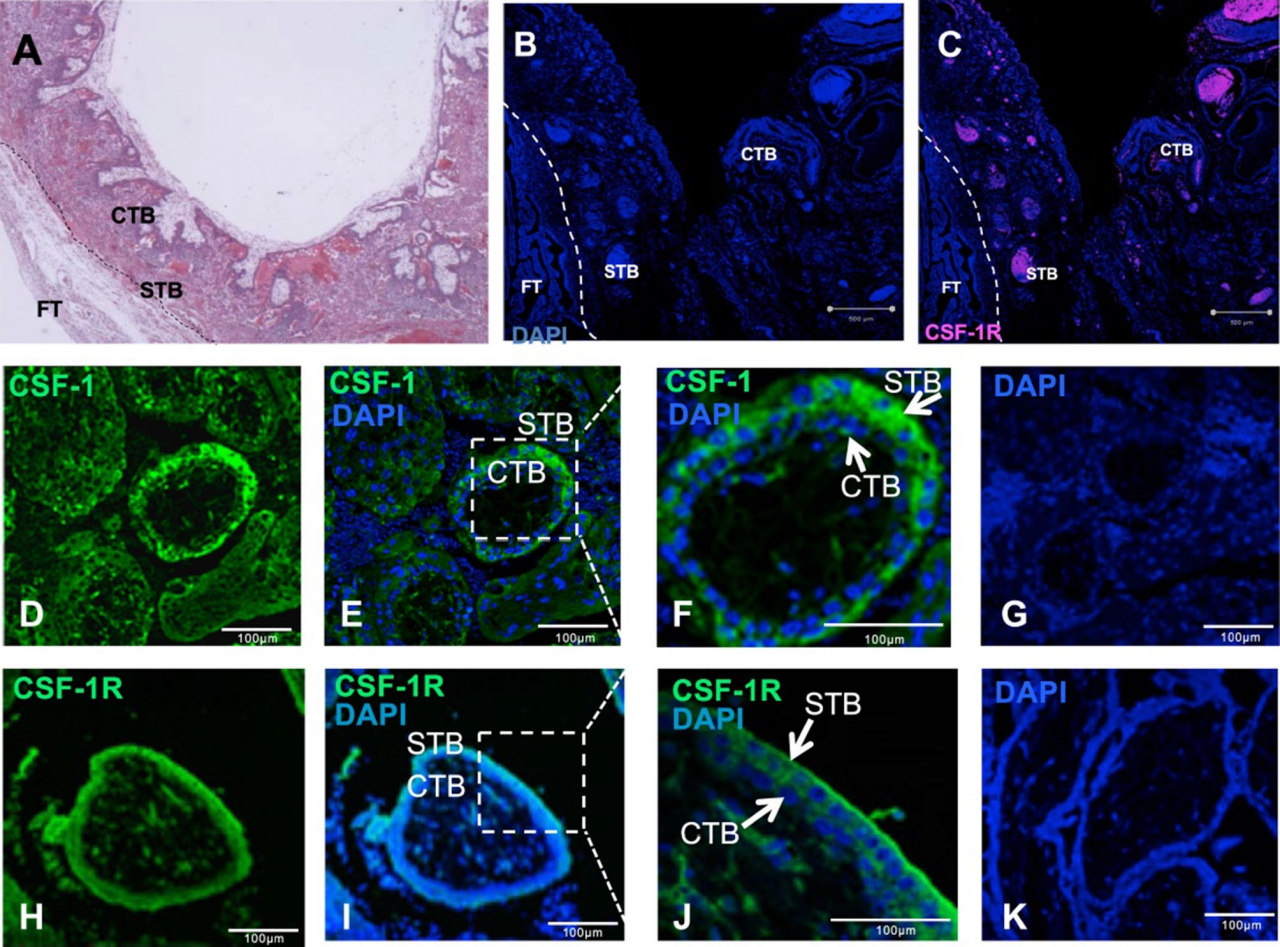
Immunolocalisation of CSF-1R and CSF-1 in tubal EP implantation sites. **(A**) Representative haematoxylin and eosin image of the ectopic implantation site in the Fallopian tube. (B) Negative control DAPI stained section (blue) showing no CSF-1R staining (red). (C) Section of human Fallopian tube ectopic implantation site immunostained for CSF-1R showing staining (red) and confirming the expression of CSF-1R. **(D**,**E)** Representative image showing immunolocalisation of CSF-1 in trophoblast invading a human FT. Immunolocalisation of CSF-1 at ectopic implantation sites is observed as green staining. **(F)** CSF-1 immunostaining (green staining highlighted by white arrows) with DAPI (blue) staining the nuclei of trophoblasts at ectopic implantation site. **(G)** negative control section with DAPI (blue) staining. **(H**,**I)** Representative image showing immunolocalisation (green) of CSF-1R in trophoblast invading a human FT. **(J)** Immunolocalisation of CSF-1R on ectopic implantation sites is shown by green staining (white arrows) with DAPI (blue) staining nuclei of trophoblasts at ectopic implantation site. (K) No immunostaining in control section with DAPIU (blue staining). *FT* Fallopian tube, *CTB* cytotrophoblast, *STB* syncytiotrophoblast. Scale bars represent 100 µm.

We examined the tubal EP implantation site in women (Fig. 3A). IF CSF-1 has effects within the trophoblasts in tubal ectopic pregnancy they would be expected to express the CSF-1 receptor. We were able to localise the CSF-1R to the tubal implantation site (Fig. 3B,C). We demonstrated the expression of CSF-1 and CSF-1R using immunohistochemistry (n = 5). CSF-1 ligand could be seen in both the syncytiotrophoblast (multinuclear tissue layer of the placenta which is adjoining to the maternal circulation) and cytotrophoblast (trophoblasts cells which are found underlying syncytiotrophoblasts that subsequently differentiate into syncytiotrophoblast) at the implantation site (Fig. 3D–G). We found that CSF-1R is expressed by the syncytiotrophoblasts with strongest staining on the outer membrane of the syncytiotrophoblast facing the maternal circulation (Fig. 3H–K). CSF-1R was also expressed by cytotrophoblasts (Fig. 3H–K). The CSF-1 signalling system is present within trophoblasts in tubal ectopic implantation sites in women.

## Discussion

Placental tissue of a normal intrauterine pregnancy is known to express high levels of CSF-1 and CSF-1R which are thought to play an important role in embryo implantation and development^19^. We therefore hypothesised that the CSF-1/CSF-1R system could be targeted for the resolution of EP. We first demonstrated that CSF-1 and CSF-1R are expressed in immortalised first trimester trophoblast cells. Then, we showed that GW2580, a specific CSF-1R antagonist, can decrease cell proliferation and migration of trophoblast cells, even following exposure to the CSF-1 ligand, and at pharmacological doses is cytotoxic. Finally, we demonstrated that CSF-1 and CSF-1R are expressed in the cytotrophoblast and syncytiotrophoblast at tubal ectopic implantation sites collected from women with EP.

We began with the knowledge that the trophoblast of an intrauterine pregnancy has by far the highest expression of CSF-1 and CSF-1R compared with any non-macrophage-lineage cell types in humans (biogps.gnf.org). The placenta relies heavily on CSF-1 signalling^20^ which has been shown to increase the proliferation of placenta-derived cells, stimulated the differentiation of cytotrophoblast in-vitro and regulates pre-implantation blastocyst cells division^21,22^. In addition, CSF-1 signalling has been shown to stimulate extravillous trophoblast cell proliferation^12^. The full role of CSF-1 during abnormal embryo implantation and in pregnancy complications is still being investigated. Low CSF-1 serum levels are associated with recurrent miscarriages^23^ and CSF-1 deficient mice demonstrated decreased embryo development^24^. Our data confirmed that CSF-1 promotes proliferation and migration of SW.71 trophoblast cells. These data are in agreement with previous findings suggesting the critical role of CSF-1 in embryo growth. Furthermore, specific inhibitor of CSF-1R, GW2580 negated the stimulatory effects of CSF-1 and could lead to cell death. We are aware that redundance of actions have been reported in the CSF family but we did not perform similar dynamic tests with other CSFs, as negative controls, to confirm the potential specificity of CSF1 action on our model. Nevertheless, our data collectively suggests that inhibiting CSF-1/CSF-1R signalling negatively affects placental cell growth, migration and survival and thus could be a potential approach to treat EP.

GW2580 selectively inhibits the tyrosine kinase domain of CSF-1R^25^ and is shown to be effective in targeting tumour invasion when used in isolation, and potentiates its efficacy when used in combination with indoximod therapy^26^. Recently, engineered bi-specific inhibitors have been developed that simultaneously target the unique combination of CSF-1 and integrins^27^, these dual-specific proteins could bind to and inhibit both CSF-1 and αvβ3 integrin and have shown superior therapeutic potential, as compared to monospecific protein therapeutics. Our recent published work has pointed a role of endometrial receptivity marker and cell adhesion protein, integrin β1 (ITGB1), in the embryo attachment within FT, as a result of past chlamydial infection^28^. We can speculate that CSF-1 and ITGB1 may function synergistically in promoting embryo implantation and growth within the FT. Taken together, our current and previous studies^29,30^ provide a rationale to develop strategies of using a combination therapy approach to treat EP. Since we have demonstrated a role of CSF-1 and ITGB1 and in EP, further investigations are warranted to develop such bi-specific inhibitors that could pave the way to discover more efficient and specific drugs to treat EP. We also believe that it would be of interest to investigate the differences in action of CSF1 on ectopic compared to eutopic pregnancies.

In conclusion, we have demonstrated that CSF-1/CSF-1R system is present at the ectopic embryo implantation site and that CSF-1 promotes trophoblast proliferation, migration and survival in-vitro. We suggest that targeting the CSF-1 system using receptor antagonists may have a novel therapeutic role in the medical management of EP. Targeted drug delivery systems, based on nanoparticles, may be additionally exploited as a new strategy for better therapeutic efficiency, along with reduction of side effects. Further in-vivo investigations are therefore warranted to decipher the role of CSF-1/CSF-1R in development of tubal EP and to determine the efficacy of CSF-1 antagonism for the treatment of EP.

## Methods

All experiments were performed in accordance with relevant guidelines and regulations.

### Cell culture

Swan.71 (RRID:CVCL_D855) (SW.71), an hTERT immortalised cell line of extravillous trophoblast cells from first trimester placenta, were maintained in Dulbecco’s Modified Eagle Medium (DMEM; Life Technologies, Paisley, UK) supplemented with 10% fetal bovine serum (FBS), 2mML-glutamine and 50ug/ml gentamycin. The cells were incubated at 37 °C in a humidified atmosphere with 5% CO_2_ levels^31^.

### Immunocytochemistry

Immunocytochemistry (ICC) was performed to demonstrate that SW.71 cells express the CSF-1 and CSF-1R. SW.71 cells were cultured on positively charged coverslips in 6-well plates until ~ 70% confluent. They were fixed using 4% paraformaldehyde for 10 min followed by methanol on ice for 2 min. Blocking was carried out with BSA and normal goat serum for 1 h. CSF-1 and CSF-1R were immunolocalised using primary antibodies conjugated with Alexa Fluor 488 against CSF-1 (Santa Cruz Biotechnology, Texas, USA, sc365779, 1:500) and CSF-1R (Santa Cruz Biotechnology, Texas, USA, sc365719, 1:500) overnight at 4 °C with blocking peptide as a negative control. The coverslips were stained with VECTASHIELD with DAPI (Vector Laboratories, Peterborough, UK) and visualised with a confocal microscope using Zen 2009 software.

### Cell proliferation assay

The effect of GW2580 and CSF-1 ligand on proliferation of SW.71 cells, was analysed using CellTiter 96 Aqueous (MTS) kit (Promega, Southampton, UK) as per the manufacturers’ recommended protocol. To optimise concentrations of treatments GW2580 (Selleckchem, Munich, Germany) was examined at 5 µM-40 µM^32^ and CSF-1 ligand (recombinant human MCSF, BioLegend, California, USA) was tested at 50–200 ng/mL^33^ time periods 24–48 h. Vehicle (DMSO) treated cells were used as the control. For the experiments reported the optimised concentration of CSF-1 was 100 ng/mL over 48 h and the concentration of GW2580 was 20–40 µM.

### ApoTox-Glo assay

Viability and cytotoxicity was measured with an ApoTox-Glo triplex assay (Promega, Southampton, UK) as per manufacturer’s protocol.

### Cell migration assay

To measure cell migration, a scratch assay was performed. SW.71 cells were seeded at a density of 150,000 per well in a 6-well plate. On the base of the plate, three vertical lines were carved under each well using a scalpel as reference points. Cells were serum starved overnight to synchronise all cell cycles, limiting the influence of proliferation, a scratch was then made horizontally across the confluent cell monolayer using a 200μL pipette tip. Culture media was replaced with increasing concentrations of GW2580, with and without 100 ng/mL CSF-1. The wells containing scratched cells were imaged at the reference points at the start of treatment and then imaged again following an optimised incubation period of 18 h with the treatments. The images were analysed using TScratch (version 1) software (https://www.cse-lab.ethz.ch/software/) in which the boundaries of the scratch are automatically delineated. TScratch software measures the percentage scratch remaining open comparing the two time points.

### Tissue resources

This study was approved by the Scotland A Research Ethics Committee (LREC 04/S1103/20) and written informed consent was taken from all study participants. FT ectopic implantation sites (n = 5) were collected from women aged 18–45 years undergoing laparoscopic salpingectomy for the treatment of tubal EP. Biopsies were transported on ice to the laboratory, fixed in 10% neutral buffered formalin overnight at 4 °C, stored in 70% ethanol, and then wax embedded for further immunohistochemical analysis.

### Immunohistochemistry

To determine the expression of CSF-1 and CSF-1R in the trophoblast invading the FT in an EP, immunohistochemistry (IHC) was performed, as previously described^34^. Briefly, 5 μm sections of ectopic implantation site were deparaffinised in xylene, rehydrated in ethanol and processed for antigen retrieval using citrate buffer in a pressure cooker for 20 min. Sections were quenched for endogeneous peroxidase in 3% H_2_O_2_ and non-specific binding sites were blocked using horse serum. Sections were incubated with primary antibodies (1 in 500) against CSF-1 (anti-CSF1, Santa Cruz Biotechnology) and CSF-1R (anti-GMCSFRα, Santa Cruz Biotechnology) overnight at 4 °C. For control sections, isotype control immunogloblins were used at the same concentration. Positive immunostaining was revealed by application of goat anti-mouse peroxidase (Dako, Ely, UK) secondary antibody followed by, signal amplification using tyramide signal amplification kit (PerkinElmer) and mounted with VECTASHIELD with Dapi (Vector Laboratories). Samples were analysed with a confocal microscope using ZEN 2009 software (https://www.softpedia.com/get/Multimedia/Graphic/Graphic-Viewers/ZEN-2009-Light-Edition.shtml).

### Statistical analysis

IHC and ICC data was analysed descriptively. Results from in-vitro assays (MTS, automated countess, ApoTox-Glo and scratch assay) are presented as the mean ± SEM and are analysed using one-way ANOVA followed by Dunn’s multiple comparisons test. All statistical analysis was performed with the use of GraphPad Prism 6.
